# Adenovirus Entry From the Apical Surface of Polarized Epithelia Is Facilitated by the Host Innate Immune Response

**DOI:** 10.1371/journal.ppat.1004696

**Published:** 2015-03-13

**Authors:** Poornima L. N. Kotha, Priyanka Sharma, Abimbola O. Kolawole, Ran Yan, Mahmoud S. Alghamri, Trisha L. Brockman, Julian Gomez-Cambronero, Katherine J. D. A. Excoffon

**Affiliations:** 1 Departments of Biological Sciences, Wright State University, Dayton, Ohio, United States of America; 2 Biochemistry and Molecular Biology, Wright State University, Dayton, Ohio, United States of America; University of Wisconsin-Madison, UNITED STATES

## Abstract

Prevention of viral-induced respiratory disease begins with an understanding of the factors that increase or decrease susceptibility to viral infection. The primary receptor for most adenoviruses is the coxsackievirus and adenovirus receptor (CAR), a cell-cell adhesion protein normally localized at the basolateral surface of polarized epithelia and involved in neutrophil transepithelial migration. Recently, an alternate isoform of CAR, CAR^Ex8^, has been identified at the apical surface of polarized airway epithelia and is implicated in viral infection from the apical surface. We hypothesized that the endogenous role of CAR^Ex8^ may be to facilitate host innate immunity. We show that IL-8, a proinflammatory cytokine and a neutrophil chemoattractant, stimulates the protein expression and apical localization of CAR^Ex8^ via activation of AKT/S6K and inhibition of GSK3β. Apical CAR^Ex8^ tethers infiltrating neutrophils at the apical surface of a polarized epithelium. Moreover, neutrophils present on the apical-epithelial surface enhance adenovirus entry into the epithelium. These findings suggest that adenovirus evolved to co-opt an innate immune response pathway that stimulates the expression of its primary receptor, apical CAR^Ex8^, to allow the initial infection the intact epithelium. In addition, CAR^Ex8^ is a new target for the development of novel therapeutics for both respiratory inflammatory disease and adenoviral infection.

## Author Summary

Respiratory viral infection is one of the leading causes of morbidity and mortality worldwide. Interventions that are able to limit viral infection will enhance human health and productivity. However, the mechanisms that control our susceptibility to viral infection and the factors that allow viral pathogens to breach the exterior epithelial barrier to initiate infection are not well understood. Here we find that adenovirus, a common cold virus and a potential gene therapy vector, uses a cellular receptor that is induced by the host innate immune response. Moreover, neutrophils, cells that are meant to protect the host in the early phase of an innate immune response, instead facilitate adenovirus infection. It has been known for over 15 years that adenovirus itself can induce an innate immune response and specifically induce host cell secretion of IL-8, a critical chemokine that attracts neutrophils to sites of infection. However, until now, it has been unclear how IL-8 induction might benefit the virus. Our data indicate that adenovirus evolved to use our innate defense system to enhance entry into the epithelium and identifies the apical adenovirus receptor as a new target that may modulate inflammatory disease.

## Introduction

Adenoviruses (AdV) are a common cause of upper and lower respiratory tract infections. Although most AdV infections are self-resolving, some may lead to acute respiratory distress syndrome, a serious and frequently fatal respiratory condition [1,2]. Epidemic AdV infections occur in closed communities, among children, and military recruits, and are most severe, often lethal, in immunosuppressed individuals [1–3]. In addition, AdV is frequently associated with exacerbation of inflammatory airway diseases such as asthma, cystic fibrosis (CF), and chronic obstructive pulmonary disease (COPD) [4–7]. No specific therapeutics exist to treat or prevent AdV infection; thus, the discovery of novel strategies to limit viral infection in susceptible populations would be an important advancement.

Human AdV is a non-enveloped double-stranded DNA virus that can be grouped into seven species (A through G), with >60 types identified [2,8]. All species, except group B, use the coxsackievirus and adenovirus receptor (CAR) as a primary receptor for cell attachment via the AdV fiber knob (FK) [9–12]. In polarized epithelial cells, CAR is found below the tight junction seal that separates the air-exposed apical surface from the basolateral surface [13]. Until recently, it was believed that AdV must breach the epithelial tight junction barrier to access CAR and initiate viral infection in the lungs [13]. It is now known that CAR has another transmembrane isoform that is able to localize at the apical surface of polarized airway epithelia and mediate AdV infection [14–16]. Whereas the basolateral isoform is composed of the first seven exons of the human *CXADR* gene (CAR^Ex7^ or hCAR1), the apical isoform occurs via splicing from a cryptic site within the seventh exon to the eighth and final exon (CAR^Ex8^). The two nearly identical proteins vary only in the last 26 (CAR^Ex7^) or 13 aa (CAR^Ex8^) of the proteins. The abundance of apical CAR^Ex8^ and the amount of AdV infection are tightly regulated by the cellular scaffold protein MAGI-1 and are increased by side-stream tobacco smoke [15,16]. Determining other cellular and environmental factors that regulate CAR^Ex8^ will provide insight into what controls the susceptibility of the host epithelium within an individual to viral infection.

The factors that predispose both healthy and immunocompromised individuals to AdV infection are complex, and likely related to the co-evolution of the host and pathogen. Similar to many other proinflammatory pathogens, AdV is a proinflammatory virus that can stimulate the secretion of proinflammatory cytokines, including interleukin-8 (IL-8), by airway macrophages and the epithelial cells within the lung epithelium [17,18]. IL-8 exposure in turn favors AdV infection of the airway epithelium [17]. How the proinflammatory cytokines enhance AdV infection remains unclear.

IL-8 is a potent neutrophil chemoattractant that initiates transepithelial migration. Previous studies have shown that basolateral CAR^Ex7^ interacts with a neutrophil surface protein, junctional adhesion molecule-like protein (JAML), and that blocking the interaction interferes with the efficiency of neutrophil transmigration [19]. The extracellular domain of CAR binds to JAML, and since the extracellular domain of CAR^Ex7^ and CAR^Ex8^ are identical, this suggests that CAR^Ex8^ might also bind to neutrophils via JAML. We hypothesized that the apical expression of CAR^Ex8^ is stimulated by IL-8 in order to function as a receptor that tethers neutrophils at the apical surface of epithelia. We further hypothesized that AdV may have co-opted this potential innate immune function of CAR^Ex8^ in order to facilitate AdV entry from the apical surface of a polarized epithelium. Finally, considering that neutrophils mainly target bacterial pathogens and antibody or complement bound molecules [18], we hypothesize that IL-8 and neutrophils contribute to AdV infection. Consistent with this, AdV is frequently isolated from patients with inflammatory respiratory diseases.

In this study, we show for the first time that IL-8 increases the protein synthesis and apical localization of CAR^Ex8^ in polarized cells via activation of the AKT/S6K pathway and inactivation of GSK3β. Apical CAR^Ex8^ tethers infiltrating neutrophils on the apical surface of polarized epithelia, a novel biological function of CAR^Ex8^, and adherent neutrophils at the apical surface enhance AdV infection. Taken together, AdV uses the host innate immune response, triggered by either invading microbes or other IL-8 stimulants entering the airway, to facilitate entry into host cells. Understanding the intricate interplay between the host innate immune system and different types of pathogens is critical in order to develop targeted therapies that prevent infection and disease progression.

## Results

### IL-8 increases airway epithelial cell susceptibility to AdV infection

**Fig 1:**
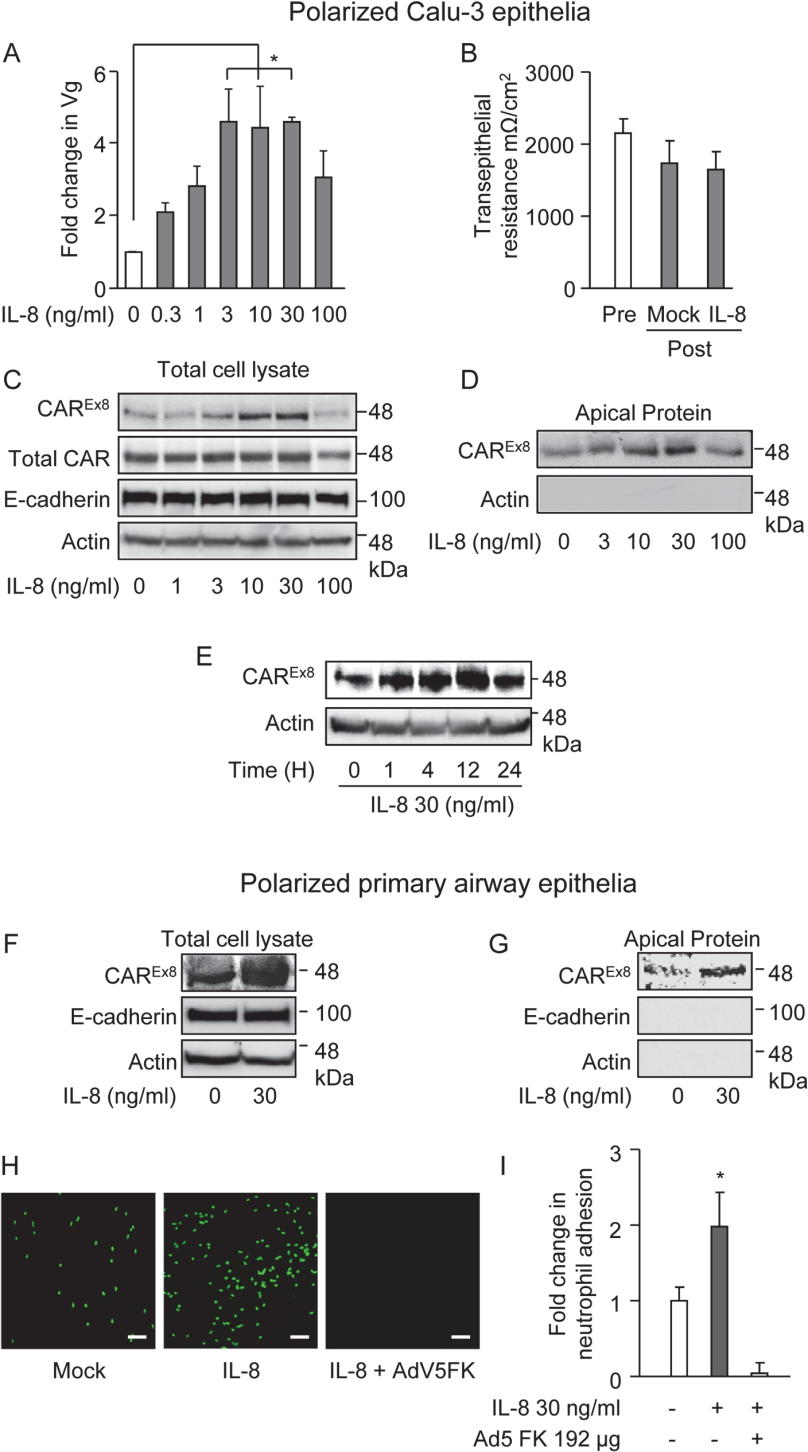
IL-8 increases the susceptibility of polarized airway epithelia to AdV entry, apical CAR^Ex8^ protein expression, and neutrophil adhesion at the apical surface. The apical surfaces of polarized A-D) Calu-3 cells or F-I) primary human airway epithelia were exposed to IL-8 for 4 h. A) Mock (0) or IL-8-exposed Calu-3 epithelia were transduced with AdV5 from the apical surface and analyzed 24 h later for the fold change in viral genomes (Vg) relative to GAPDH by qPCR. B) TER before or after IL-8 (30 ng/ml) exposure. C) Western blots for CAR^Ex8^, total CAR, actin, and E-cadherin protein expression in lysates or D) CAR^EX8^ and actin after apical surface-specific biotinylation. E) CAR^Ex8^ and actin protein expression in lysates from Calu-3 cells exposed to IL-8 for different lengths of time. The apical surface of polarized primary airway epithelial cells were exposed to IL-8 and F) CAR^Ex8^, actin, and E-cadherin protein expression in lysates or G) after apical surface-specific biotinylation. H) Polarized primary human airway epithelia were either mock or IL-8 treated for 4 h. Cells were then either untreated or treated with purified AdV5 FK, as indicated, followed by an adhesion assay with primary neutrophils stained with calcein green. Bound neutrophils were imaged using fluorescence microscopy (10X lens, white bar = 150 μm) and I) quantified using Metamorph software. Error bars represent the SEM from three independent experiments: *p < 0.05, A and B by one-way ANOVA or I, IL-8 treatment versus untreated or FK treated.

To investigate the effect of IL-8 on AdV infection in polarized epithelia, we first used polarized Calu-3 airway epithelial model cells. Polarized Calu-3 epithelia were treated with increasing concentrations of IL-8 (0–100 ng/ml (0–12.5 nM)) for 4 h, followed by apical infection with recombinant, replication-defective, AdV type 5 (AdV5). Quantitative PCR (qPCR) analysis for AdV5 genomes (Vg) was performed by determining the copy number of the AdV5 *hexon* gene relative to a cellular housekeeping gene after DNA extraction. QPCR showed that AdV entry was increased in response to IL-8 treatment in a dose-dependent manner (Fig. 1A). Viral entry reached its maximum and plateaued at 3, 10 and 30 ng/ml of IL-8, with ∼5-fold increase in Vg when compared to control (0 ng/ml IL-8; p<0.05), followed by a decrease at 100 ng/ml. However, there was no significant change in the transepithelial resistance (TER) indicating that the effect of IL-8 on viral entry was not due to decreased integrity of the epithelial junctions (Fig. 1B). These data suggest that the increase in epithelial susceptibility to AdV entry upon IL-8 exposure may be due to specific cellular effects, such as increased primary receptor expression at the apical surface of the polarized epithelium.

### IL-8 increases the protein expression and the localization of apical CAR^Ex8^

The primary receptor for AdV5 is CAR; therefore, we investigated the expression of CAR in the presence of IL-8. In particular, we examined the expression of the apical isoform of CAR, CAR^Ex8^, since it is known to be present at the air-exposed surface of airway epithelia [14]. Polarized Calu-3 cells were treated with IL-8 at varying concentrations (Fig. 1C, D) and for varying time points (Fig. 1E). IL-8 increased the expression of CAR^Ex8^ in both a concentration and time-dependent manner. IL-8 had its maximal effect on CAR^Ex8^ expression at 30 ng/ml (Fig. 1C, D). By contrast, IL-8 did not affect the amount of total CAR, which is predominantly composed of the basolaterally-sorted CAR^Ex7^ isoform [14,15]. IL-8 also did not affect the junction-adhesion protein E-cadherin or actin (loading control) (Fig. 1C). Consistent with increased CAR^Ex8^ protein levels in lysates, apical-surface specific biotinylation assays showed that CAR^Ex8^ localization at the apical surface increased in response to IL-8 in a dose-dependent manner, with a maximum increase at 30 ng/ml (Fig. 1D). By contrast, no biotinylated cytosolic actin was detected. IL-8 had its maximal effect on CAR^Ex8^ protein expression between 4–12 h at 30 ng/ml and returned to baseline levels within 24 h (Fig. 1E). To investigate acute effects on the epithelium, further experiments were carried out with 30 ng/ml of IL-8 for 4 h. Well-differentiated primary airway epithelia obtained from healthy human donors were used to validate the results found in Calu-3 epithelia. Similar to Calu-3 epithelia, apical treatment with 30 ng/ml of IL-8 for 4 h resulted in a robust increase in CAR^Ex8^ protein expression (Fig. 1F) and apical localization (Fig. 1G). As expected, the protein levels of the basolateral junctional-adhesion protein E-cadherin and cytosolic actin did not change (Fig. 1F) and were not detected upon apical surface-specific biotinylation (Fig. 1G).

### IL-8 treatment increases neutrophil adhesion on the apical surface of polarized primary human airway epithelial cells

Neutrophils at the apical surface of an epithelium play a critical role in pathogen clearance [20]. Since CAR^Ex8^ protein expression in polarized epithelia is activated by the neutrophil chemoattractant IL-8 and since the extracellular region of epithelial CAR^Ex7^ that binds JAML on the surface of neutrophils is identical to CAR^Ex8^, we hypothesized that CAR^Ex8^ has a role in tethering neutrophils to the apical surface of the epithelium. To test this hypothesis, polarized primary human airway epithelia (Fig. 1H and I) or polarized Calu-3 cells (S1A–S1B Fig) were pre-stimulated with IL-8. Neutrophils isolated from the healthy human donors were fluorescently labelled and added to the apical surface of the epithelia for a neutrophil adhesion assay. Neutophil binding was determined by total fluorescence integrated density over 5–10 images per condition. IL-8 treatment resulted in a significant 2–2.5 fold increase in the adhesion of primary neutrophils. Neutrophil adhesion was completely blocked when the IL-8 pre-stimulated polarized epithelia were pre-treated with AdV5 fiber knob (FK), the capsid protein that binds to the extracellular domain of CAR with high affinity (Fig. 1H and I). These data indicate that CAR^Ex8^ is an important component of IL-8 stimulated apical neutrophil adhesion.

### Induction of CAR^Ex8^ protein expression significantly increases susceptibility to AdV entry and transduction

**Fig 2:**
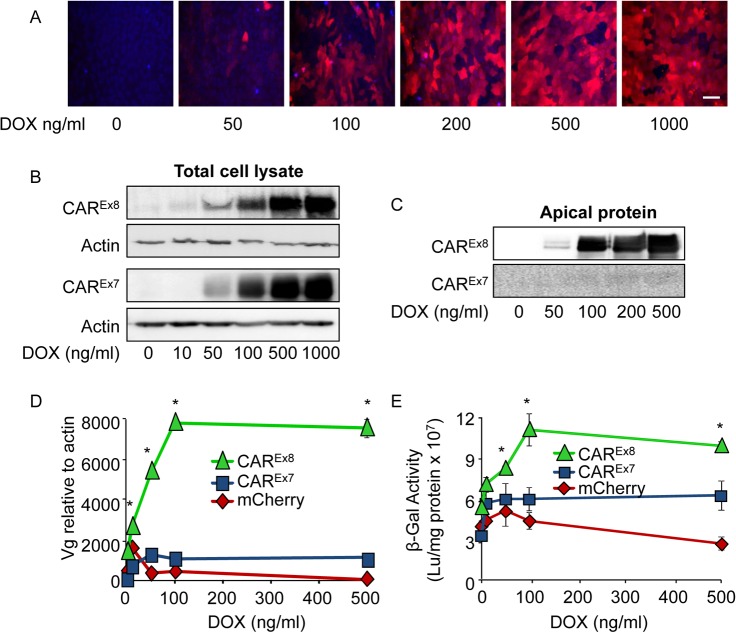
Induction of CAR^Ex8^ expression increases the susceptibility of polarized epithelia to AdV entry and transduction. A) MDCK-mCherry cells either mock (0) or DOX treated for 24 h were imaged using fluorescence microscopy (20X, white bar = 30 μm). Hoechst 33342 staining (blue) indicates cellular nuclei. B) Flag-CAR^Ex8^, Flag-CAR^Ex7^ protein expression was analyzed in lysates from MDCK-CAR^Ex8^ and-CAR^Ex7^ cells, respectively, after mock (0) or DOX induction. C) Apical surface-specific biotinylation of mock- (0) or DOX-induced polarized-MDCK-CAR^Ex8^ or-CAR^Ex7^ cells analyzed by Western blot using an anti-FLAG-tag Ab. D) Polarized MDCK-stable cells were treated with increasing concentrations of DOX for 24 h, transduced with AdV5-βGal from the apical surface for 1 h, and analyzed 24 h post-infection for viral entry by qPCR (viral genomes, Vg) or E) viral transduction via β-gal activity. Error bars represent the SEM from three independent experiments; *p < 0.05 by two-way ANOVA.

To confirm that CAR^Ex8^ is responsible for increased AdV5 transduction and that CAR^Ex8^ tethers neutrophils at the apical surface of polarized epithelia, model epithelial cells stably expressing CAR^Ex8^ under a Doxycycline (DOX) inducible promoter were generated. Control cells stably expressing CAR^Ex7^ or mCherry were also generated from the same parental Tet-on MDCK cell line. MDCK cells were chosen because these cells are well characterized, grow quickly, and polarize rapidly into an epithelium with an expected distribution of cellular proteins [21–23]. Polarized epithelia from cell lines derived from single-cell clones with stable integration of FLAG-tagged CAR^Ex8^, FLAG-tagged CAR^Ex7^, or mCherry, under the DOX sensitive P_Tight_ promoter were characterized and compared in the absence of DOX. Clones were selected that had similar growth and polarization characteristics, including the ability to form tight junctions, distribution of apical, basolateral, and tight junction proteins, and polarity of baseline AdV5 transduction. MDCK cells stably expressing mCherry, FLAG-tagged CAR^Ex8^, or FLAG-tagged CAR^Ex7^, demonstrated a DOX-dose dependent increase of mCherry fluorescence (Fig. 2A), or CAR^Ex8^ or CAR^Ex7^ protein levels relative to actin (Fig. 2B). To confirm the polarity of protein expression with the polarized MDCK epithelium, apical surface-specific biotinylation was performed. In contrast to CAR^Ex7^ in MDCK-CAR^Ex7^ epithelia, CAR^Ex8^ protein was detected at the apical surface of MDCK-CAR^Ex8^ epithelia at low doses of DOX and expression was saturated above 100 ng/ml of DOX (Fig. 2C).

To characterize the susceptibility of the MDCK stable cell lines to AdV infection, cells were polarized and infected with AdV5-β-Gal from the apical surface. Data from quantitative PCR (viral genomes, Vg; Fig. 2D) and transduction (β-Gal expression; Fig. 2E) showed a dose-dependent increase in adenoviral entry into MDCK-CAR^Ex8^ epithelia exposed to low levels of DOX, which was not observed in MDCK-CAR^Ex7^ and MDCK-mCherry epithelia. These data show that apical AdV entry and transduction is highly sensitive to the induction of apical CAR^Ex8^ expression in polarized epithelia. Consistent with the findings by Western blot (Fig. 2B, C), the MDCK-CAR^Ex8^ epithelia demonstrated a plateau in viral genome entry and transduction above 100 ng/ml DOX treatment, suggesting that there may be cellular limits to the amount of CAR^Ex8^ expressed within a cell, the amount of CAR^Ex8^ available at the apical surface, or limitations to viral entry at the apical surface.

### CAR^Ex8^ tethers neutrophils at the apical epithelial surface

**Fig 3:**
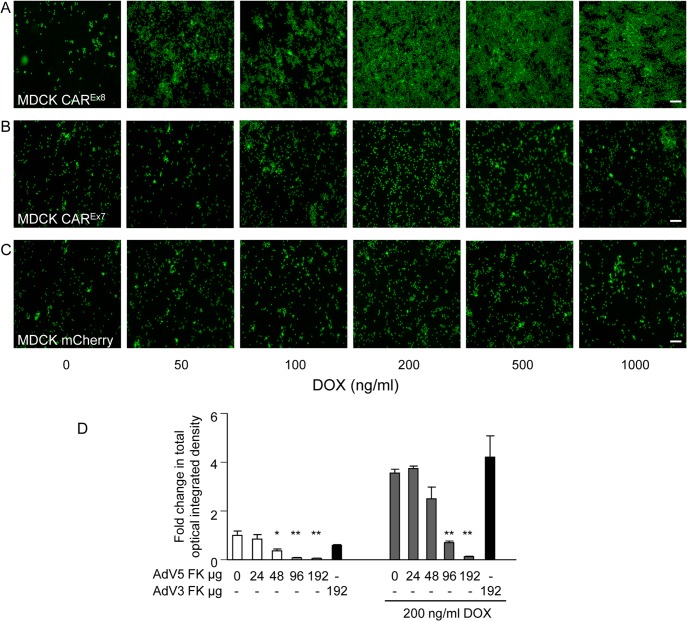
Apical CAR^Ex8^ protein expression increases apical neutrophil adhesion that is sensitive to AdV5 FK. A neutrophil adhesion assay was performed on mock (0) or DOX-induced A) MDCK-CAR^Ex8^, B)-CAR^Ex7^, or C)-mCherry cells. Adhered neutrophils (green) on the surface of the epithelial cells were captured by fluorescence microscopy (10X; white bar = 100 μm). D) MDCK-CAR^Ex8^ either mock (0) or DOX-induced, as indicated, were treated with AdV5 FK or AdV3 FK immediately prior to performing the neutrophil adhesion assay. Adhered neutrophils were captured by using fluorescence microscopy and quantitated using Metamorph software. Images and quantitation are representative of 5–10 images from at least 3 separate experiments. Error bars represent the SEM from three independent experiments; *p < 0.05 or **p < 0.01 by one-way ANOVA. White bar, 100 μM.

Since IL-8 induces CAR^Ex8^ protein expression and increases neutrophil retention at the apical surface of polarized epithelia (Fig. 1), we hypothesized that induction of CAR^Ex8^ protein expression in the absence of IL-8 would be sufficient to increase the binding of neutrophils at the apical surface of polarized epithelia. To test this, polarized MDCK-CAR^Ex8^,-CAR^Ex7^ and-mCherry epithelial cells were induced with increasing concentrations of DOX for 24 h and a neutrophil adhesion assay was performed. Increasing apical CAR^Ex8^ protein levels in MDCK-CAR^Ex8^ epithelia correlated directly with increased neutrophil adhesion on the epithelial cell surface (Fig. 3A). By contrast, MDCK-CAR^Ex7^ and-mCherry DOX-induced epithelia only showed baseline neutrophil adhesion (Fig. 3B, C). These data suggest that CAR^Ex8^ is able to tether neutrophils at the apical epithelial cell surface. To confirm that this was a CAR^Ex8^-mediated effect, purified AdV5 fiber knob (FK), which has a 500–1000 fold higher affinity for the overlapping CAR-JAML or CAR-CAR binding site [24–26], was used to compete with the putative interaction between epithelial apical CAR^Ex8^ and neutrophil JAML. AdV5 FK decreased neutrophil adhesion in a dose-dependent manner in both mock and DOX-induced MDCK-CAR^Ex8^ cells, including a complete block of neutrophil adhesion at the highest concentration of AdV5 FK (Fig. 3D). In contrast, FK from AdV3, a group B AdV that does not use CAR as a primary receptor [27], did not block neutrophil adhesion (Fig. 3D). Taken together, these data show that CAR^Ex8^ tethers neutrophils at the apical epithelial cell surface and that AdV may potentially be able to out-compete neutrophils to bind apical CAR^Ex8^.

### CAR^Ex8^ tethers infiltrating neutrophils at the apical epithelial cell surface

**Fig 4:**
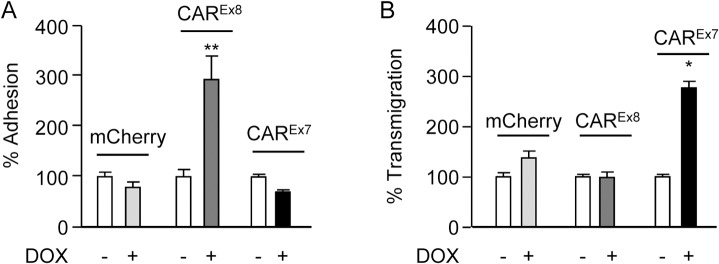
Apical CAR^Ex8^ protein expression increases apical adhesion of infiltrating neutrophils. Neutrophil transmigration assays were performed in the basal-to-apical direction in MDCK stable cells exposed to the neutrophil chemoattractive peptide fMLP on the apical surface. A) % neutrophil adhesion and B) % neutrophil transmigration were quantitated by measuring the fluorescence intensity of fluorescently-labeled neutrophils imaged by fluorescence microscopy. Error bars represent the SEM from three independent experiments; *p < 0.05 or **p < 0.01 by one-way ANOVA.

Next, we sought to determine the fate of neutrophils that transmigrate from the physiologically relevant basal surface to the apical surface in the presence or absence of DOX-induced CAR^Ex8^. To do this, fluorescently-labeled neutrophils were added to the basal surface of epithelia and stimulated to transmigrate to the apical surface by adding the neutrophil chemoattractive bacterial peptide fMLP to the apical surface. Two populations of cells were quantified: 1) neutrophils that transmigrated through but remained adhered to the apical epithelial surface and 2) neutrophils that completely transmigrated through and detached from the epithelium (Fig. 4A, B respectively). DOX-induced MDCK-CAR^Ex8^ epithelia retained ∼3 times as many transmigrated neutrophils on the apical surface as compared to uninduced MDCK-CAR^Ex8^ epithelia, and MDCK-CAR^Ex7^, or-mCherry epithelia regardless of DOX-induction or not (Fig. 4A). In contrast, ∼3 times as many neutrophils transmigrated through induced MDCK-CAR^Ex7^ epithelia relative to all other conditions (Fig. 4B). This is consistent with the known role for basolateral CAR^Ex7^ in facilitating neutrophil transepithelial migration [19]. These data confirm that CAR^Ex8^ is able to enhance the adhesion of transmigrating neutrophils at the apical surface and also indicate that each CAR isoform plays a distinct role in neutrophil recruitment.

### Tethered neutrophils augment viral entry into polarized epithelia

**Fig 5:**
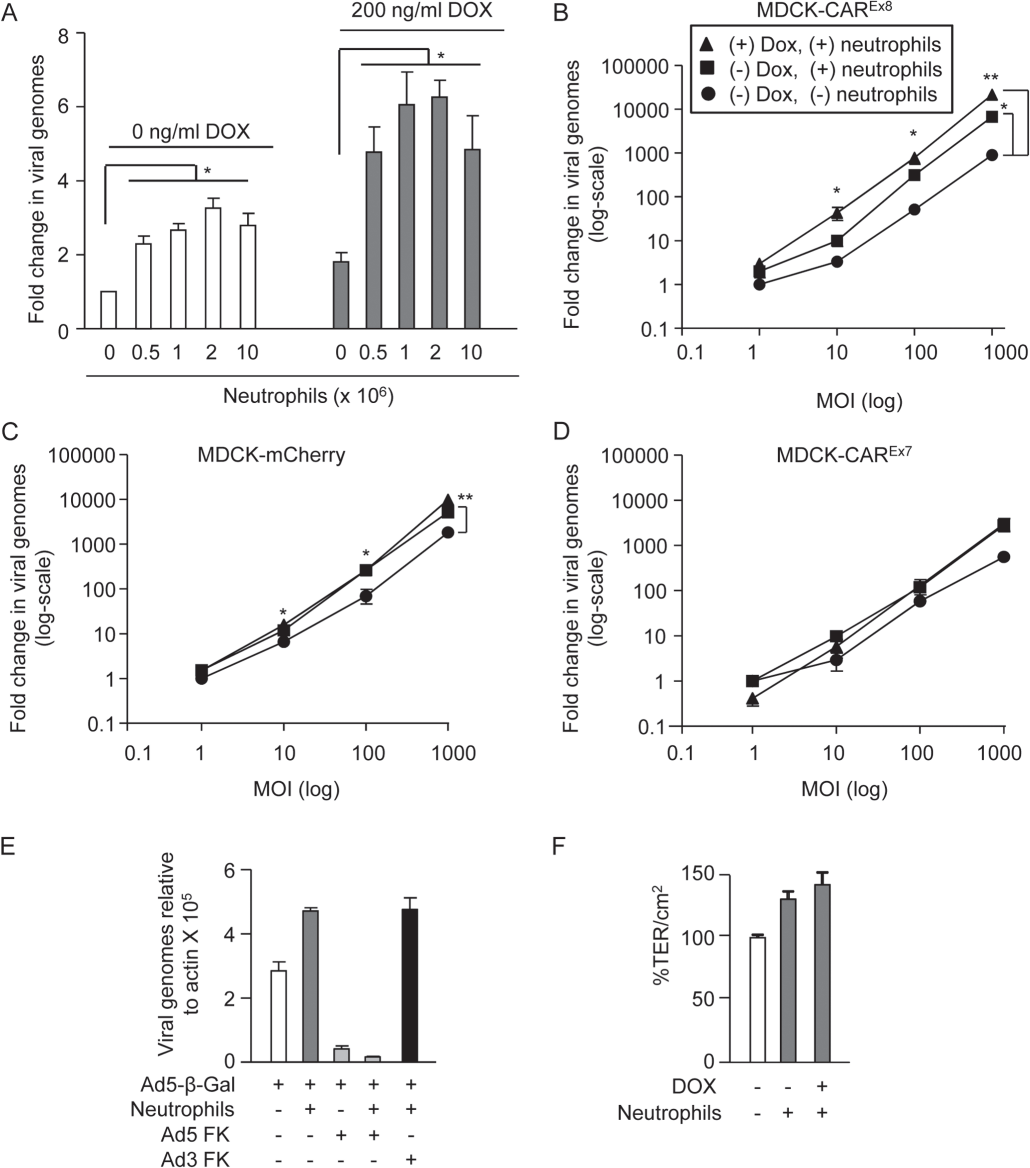
Neutrophils adhered to the apical surface of polarized-MDCK cells augment AdV entry without decreasing the TER. A) MDCK-CAR^Ex8^ cells were either mock- or DOX-induced. A neutrophil adhesion assay was performed with increasing numbers of neutrophils, as indicated. Immediately post-neutrophil adhesion, MDCK-CAR^Ex8^ epithelia were infected with AdV5-β-gal for 1 h from the apical surface. 24 h later, viral entry was determined by qPCR analysis. Fold change in viral genomes, relative to AdV5-βGal entry in the absence of DOX and neutrophils, is shown. AdV entry from the apical surface was quantitated by qPCR analysis of polarized B) MDCK-CAR^Ex8^ C) MDCK-mCherry and D) MDCK-CAR^Ex7^ cells that were uninduced (circles), uninduced with adhered neutrophils (squares), or induced with DOX for 24 h prior to neutrophil adhesion (triangles). E) AdV5-β-gal entry from the apical surface of MDCK-CAR^Ex8^ epithelia in the presence or absence of neutrophils and AdV5 FK or AdV3 FK. F) TER of mock- or Dox-induced MDCK-CAR^Ex8^ epithelia was measured in the presence or absence of neutrophils. Error bars represent standard error of the mean (SEM) from three independent experiments. No significant difference was detected by one-way ANOVA. Error bars represent the SEM from three independent experiments; *p < 0.05 or **p < 0.001 by one-way ANOVA and Bonferroni post hoc test.

Neutrophils are part of the innate immune system and the first cells recruited to sites of injury or pathogenic invasion. In order to understand the contribution of neutrophils bound to the apical surface to AdV infection, increasing amounts of primary human neutrophils (0–1 x 10^7^ cells) were allowed to bind to the apical surface of mock-induced or DOX-induced polarized MDCK-CAR^Ex8^ epithelia. Unbound neutrophils were removed by washing and AdV5-β-Gal was added to the apical surface for 1 h at 37°C. Viral entry was quantified 24 h later by qPCR. Neutrophils enhanced AdV entry by approximately 2–3 fold (Fig. 5A, white bars) and, consistent with a significant increase in neutrophil binding, AdV entry was increased by an additional 2-fold when CAR^Ex8^ expression was induced with DOX (Fig. 5A, grey bars). To determine whether neutrophil-enhanced AdV entry was dependent on viral dose, 2 X 10^6^ neutrophils were allowed to bind CAR^Ex8^, or control CAR^Ex7^ and mCherry, mock-induced or DOX-induced epithelia followed by apical transduction with increasing MOI of AdV5-β-Gal (Fig. 5B-D). To control for baseline infection, mock-induced epithelia having no neutrophils were also similarly infected with AdV5-β-Gal. Neutrophils increased apical AdV entry by 3–10-fold in MDCK-CAR^Ex8^ epithelia at all MOI (Fig. 5B). Except at MOI 1, this increase was further amplified by at least 3 fold in the presence of DOX, indicating that both neutrophils and the level of apical CAR^Ex8^ play a major role in AdV entry. In the case of uninduced mCherry epithelia (Fig. 5C), neutrophils significantly increased AdV entry in a similar manner as uninduced MDCK-CAR^Ex8^ cells, while MDCK-CAR^Ex7^ epithelia followed this trend (Fig. 5D). However, no significant change in AdV entry occurred in the presence of DOX indicating the importance of CAR^Ex8^ expression. Taken together, these data show that neutrophils facilitate viral entry into the polarized MDCK epithelium, particularly upon induction of apical CAR^Ex8^ expression. To confirm that the effect of neutrophils on AdV entry depends on CAR, polarized MDCK-CAR^Ex8^ cells were treated with either AdV5 FK or AdV3 FK, followed by neutrophil adhesion and infection with AdV5-β-Gal. We observed that AdV5 FK blocked AdV5-β-Gal entry by ∼7-fold in the absence of adhered neutrophils (p<0.0001, Fig. 5E). In the presence of adhered neutrophils, AdV5 FK, but not AdV3 FK, blocked AdV5-β-Gal entry by ∼25-fold (p<0.0001). The difference in fold change reflects the increased AdV-β-Gal entry in the presence of neutrophils. These results indicate that neutrophils promote adenoviral entry via CAR^Ex8^. To further confirm that neutrophils were not simply disrupting the epithelial tight junction, TER was measured in the presence or absence of apically adhered neutrophils. Interestingly, a trend towards increased transepithelial resistance was observed when compared to MDCK-CAR^Ex8^ cells without neutrophils (Fig. 5F). A lack of tight junction disruption is consistent with the evidence that increasing the basolateral CAR^Ex7^ isoform does not further augment viral infection in the presence of neutrophils (Figs. 2B and 5D).

### IL-8 regulates CAR^Ex8^ expression by post-transcriptional mechanisms

**Fig 6:**
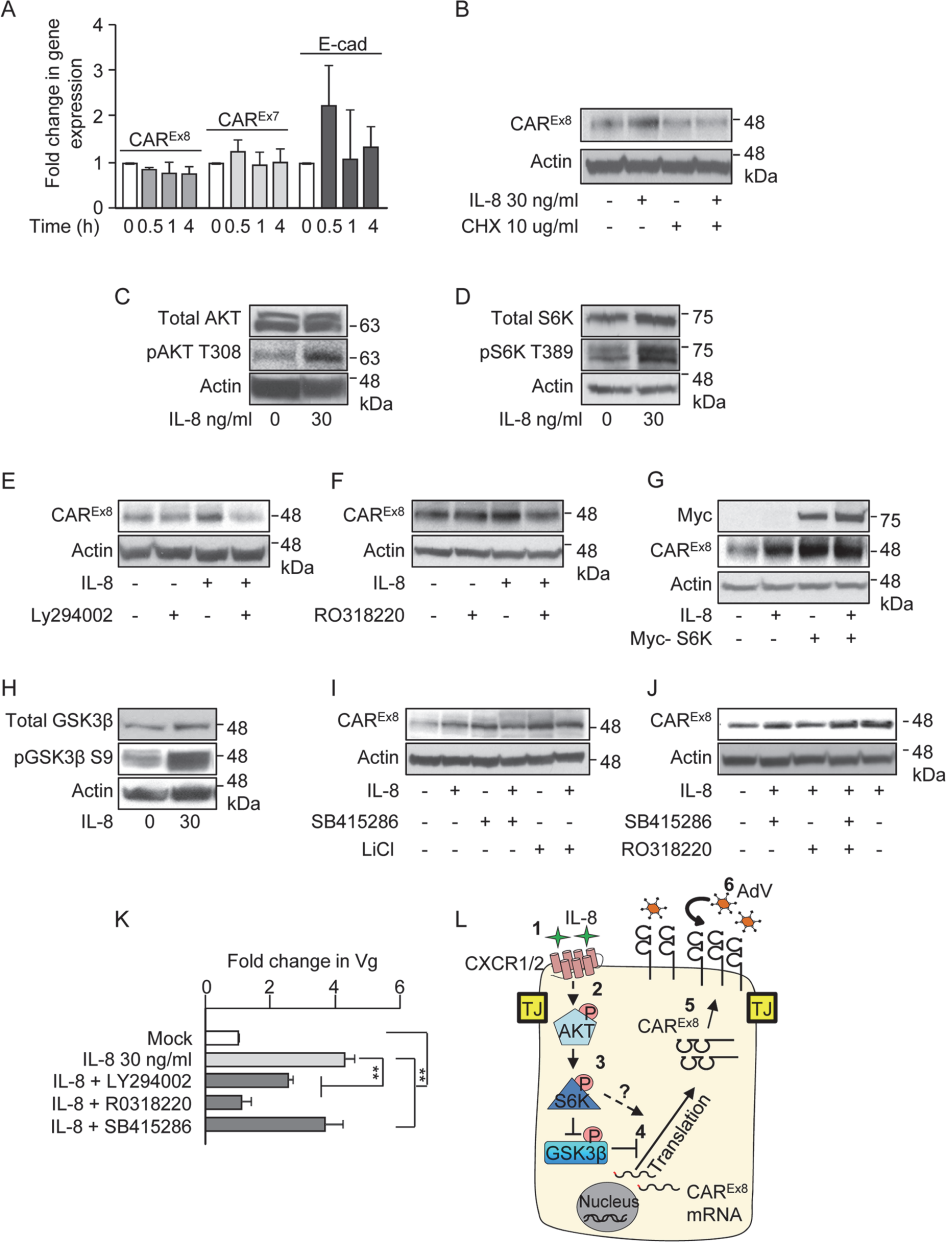
IL-8 activates AKT/S6K and inactivates GSK3β to increase CAR^Ex8^ protein synthesis and AdV entry. A) The apical surfaces of polarized primary airway epithelial cells were either mock (0, white bars) or IL-8 (30 ng/ml, gray bars) treated for the indicated time and analyzed for CAR^Ex8^, CAR^Ex7^, or E-cadherin (E-cad) gene expression by qPCR, relative to GAPDH. B) The apical surfaces of polarized primary airway epithelial cells were mock (0) or IL-8 treated in the presence or absence of cycloheximide (CHX) and lysates were analyzed for CAR^Ex8^ and actin protein expression. Activation state of C) AKT, D) S6K and H) GSK3β was analyzed after IL-8 treatment by probing for the pAKT T308, pS6K T389, and pGSK3β S9 respectively. Lysates from polarized cells treated with IL-8 in the presence or absence of chemical inhibitors for E) AKT (Ly294002, 30 μM), F) S6K (RO3118220, 300 nM), I) GSK3β (SB415286, 45 μM, or LiCl, 10 mM), or J) a combination of S6K (RO3118220, 300 nM) and GSK3β (SB415286, 45 μM) were investigated for CAR^Ex8^ and actin protein expression. G) Polarized cells were either transfected or not with myc-tagged S6K plasmid prior to mock (0) or IL-8 treatment followed by the analysis of CAR^Ex8^ and actin protein expression from cell lysates. K) Polarized cells exposed to IL-8 in the presence or absence of the indicated chemical inhibitors for 4 h were washed and transduced with AdV5-βGal for 1 h. Genomic DNA was isolated 24 h post-transduction and analyzed for the fold change in Vg normalized to GAPDH and relative to mock. Error bars represent the SEM from three independent experiments: **p < 0.001 by one way ANOVA and Bonferroni post hoc test. L) A schematic of a predicted model showing that 1) IL-8 binds to the IL-8 receptor (CXCR1/2) and 2) activates AKT. 3) Activated AKT (pAKT T308) further activates S6K (pS6K T389) and 4) activated AKT directly and/or via inhibition of GSK3β (pGSK3β S9) stimulates CAR^Ex8^ protein synthesis. 5) Newly synthesized CAR^Ex8^ traffics to the apical surface and 6) can mediate apical AdV infection.

To determine the mechanism by which IL-8 stimulates endogenous CAR^Ex8^ protein expression, transcription of CAR^Ex8^-specific mRNA was first investigated in polarized Calu-3 cells (S2A Fig) and in polarized primary human airway epithelia (Fig. 6A). CAR^Ex8^, CAR^Ex7^, and E-cadherin mRNA levels did not significantly change within 4 h of IL-8 treatment (Fig. 6A) or when treated with different IL-8 concentrations (S2A Fig) indicating that the increase in CAR^Ex8^ was by post-transcriptional mechanisms. Accordingly, co-treatment of polarized Calu-3 (S2B Fig) or primary human airway epithelia (Fig. 6B, quantitated in S3A Fig) with IL-8 and the protein synthesis inhibitor cycloheximide (CHX) abolished the IL-8 mediated increase in CAR^Ex8^ expression indicating that IL-8 acutely stimulates *de novo* CAR^Ex8^ protein synthesis.

### IL-8 activates AKT and S6K to upregulate CAR^Ex8^ protein expression

We then asked which signaling proteins downstream of IL-8 stimulation are involved in the IL-8-mediated post-transcriptional increase of CAR^Ex8^. It is known that IL-8 activates AKT, leading to the downstream activation of ribosomal S6 protein kinase (S6K) and protein translation [28]. Consistent with this, a robust activation of both AKT (phospho-AKT-T308; Figs. 6C and S3B) and S6K (phospho-S6K T389; Figs. 6D and S3C) was observed in response to IL-8 treatment. To determine whether the IL-8-mediated increased CAR^Ex8^ protein expression is downstream of AKT and S6K activation, polarized epithelia were incubated with IL-8, chemical inhibitors for AKT (Ly294002; Figs. 6E and S3D) or S6K (RO318220; Figs. 6F and S3E), or a combination of IL-8 and each inhibitor. Whereas IL-8 increased CAR^Ex8^ protein expression and each inhibitor alone did not affect CAR^Ex8^ protein expression, the inhibitors were able to block the IL-8-mediated increase in CAR^Ex8^ protein levels (Figs. 6E, F, S3D, S3E). To further test the role of S6K in the regulation of CAR^Ex8^ protein expression, Myc-tagged S6K was expressed in Calu-3 cells by plasmid transfection (Figs. 6G and S3F). Overexpression of Myc-S6K increased CAR^Ex8^ protein expression to a level similar to IL-8 treatment. Interestingly, we did not observe an additive effect between Myc-S6K and IL-8 treatment indicating that S6K is a major regulator of CAR^Ex8^ protein translation, and potentially that the amount of CAR^Ex8^ mRNA is limited. Taken together, these data show that IL-8 regulates the expression of CAR^Ex8^ via the AKT/S6K pathway.

### IL-8 inactivates GSK3β to upregulate CAR^Ex8^ protein expression

GSK3β is a multifunctional, constitutively active kinase that plays a role in multiple cellular pathways, including post-transcriptional regulation of protein expression [29]. We have previously shown that GSK3β negatively regulates CAR^Ex8^ expression and inhibition of GSK3β increases CAR^Ex8^ protein levels [16]. Although to our knowledge, GSK3β is not a known target of IL-8 signaling pathways, we investigated the activity of GSK3β upon IL-8 treatment. IL-8 treatment of polarized epithelia increased the inactivated form of GSK3β (phospho-GSK3β-S9; Figs. 6H and S3G). To further validate the involvement of GSK3β inhibition in the increase of CAR^Ex8^ protein expression, epithelia were treated with GSK3β inhibitors (SB415286 or LiCl) for 4 h in the presence or absence of IL-8. We observed that both GSK3β inhibitors increased CAR^Ex8^ protein expression to a level similar to that observed with IL-8 treatment (Figs. 6I and S3H). No further increase in CAR^Ex8^ protein expression was observed with the addition of GSK3β inhibitor to IL-8. Taken together, these data indicate that IL-8 regulates CAR^Ex8^ protein expression by inhibiting GSK3β.

### GSK3β inhibition is dominant over S6K inhibition in the presence of IL-8

Since inhibition of S6K reverses the stimulatory effect of IL-8 on CAR^Ex8^ protein expression (Fig. 6F) and inhibition of GSK3β augments CAR^Ex8^ protein expression to the same extent as IL-8 treatment (Fig. 6I), we asked whether these two signaling proteins lay in the same or different pathways. Polarized cells were treated with IL-8 while inhibiting GSK3β (SB415286) and S6K (RO318220), individually and combined, and compared to mock treated cells. The data showed an increase in CAR^Ex8^ protein levels upon treatment with the combination of IL-8, GSK3β inhibitor and S6K inhibitor (Figs. 6J, lane 4, and S3I) indicating that blocking GSK3β relieves the inhibition of CAR^Ex8^ protein translation that is either downstream of or independent from the S6K pathway.

### AKT, S6K, and GSK3β affect apical AdV infection of polarized epithelia

To determine whether the effects of the above pathways on CAR^Ex8^ protein expression alter AdV infection, polarized epithelia were treated with IL-8 alone or in the presence of inhibitors for AKT (Ly294002), S6K (RO318220), or GSK3β (SB415286) for 4 h. Inhibitors and IL-8 were removed and AdV5β-Gal was added to the apical surface for 1 h at 37°C. Viral entry was quantified by qPCR 24 h later (Fig. 6K). Consistent with decreased CAR^Ex8^ protein expression upon inhibition of AKT and S6K, viral entry decreased and S6K inhibition completely reversed the effect of IL-8 stimulation (first four bars, Fig. 6K). Consistent with the finding that GSK3β inhibition does not further increase CAR^Ex8^ protein expression upon IL-8 treatment, viral entry was identical with or without GSK3β inhibitor (Fig. 6K, last bar compared to second bar). Given the above data, and taken together with current literature, we propose the model that activation of AKT by IL-8 exposure activates S6K which either directly, or via inactivation of GSK3β, is able to augment translation of the pool of mRNA present for CAR^Ex8^ (Fig. 6L).

## Discussion

Viruses are sophisticated biological entities that can often initially infect epithelial cells without damaging the tight junction barrier integrity [13,30]. In the absence of preexisting immunity, viruses have evolved mechanisms to avoid inciting a robust inflammatory response and epithelial damage until replication has occurred so that progeny virions can co-opt the inflammatory response to enhance viral dissemination. Many inflammatory factors have been shown to modulate viral infections [31] and AdV infections are common in patients with inflammatory respiratory diseases such as COPD, CF, and asthma [4–7]. In this study, we show that the level of the apical AdV receptor, CAR^Ex8^, is a major predictor of the susceptibility of an epithelium to AdV infection and that IL-8 and neutrophils, components of the innate immune system, enhance AdV entry.

The proinflammatory cytokine IL-8 has previously been shown to increase the susceptibility of an airway epithelium to AdV infection potentially by translocation of an AdV5 co-receptor, αvβ3 integrin, to the apical surface of airway epithelia [17]. Consistent with this, we found that IL-8 exposure increased the levels of AdV5 co-receptor β_1_ integrin [32] at the apical surface of Calu-3 cells (S4A Fig). Several co-receptors have been described for AdV5 and integrins have specifically been shown to facilitate adenoviral endocytosis and endosomal escape [32–34]. However, CAR is the primary receptor that mediates efficient virus attachment, a crucial step that occurs prior to integrin binding and viral entry [9,32–35]. We show for the first time that physiologically relevant levels of IL-8 stimulate the protein expression and the apical localization of the primary apical AdV receptor, CAR^Ex8^, in polarized human airway epithelia. Consequently, this enhances Ad5 FK-sensitive AdV infection from the apical surface of the epithelium (Figs. 1A and 5E). Interestingly, the IL-8-mediated effect was reduced at 100 ng/ml concentration. This finding is consistent with physiological studies that have demonstrated a bell-shaped dose response to IL-8 for neutrophil migration due to receptor saturation and desensitization [36]. It is also possible that IL-8-mediated signaling may undergo negative feedback to inhibit IL-8 signaling by downregulating the IL-8 receptor [37]. IL-8 appears to be CAR^Ex8^-specific since it did not affect the expression of total CAR, which is predominantly CAR^Ex7^ [14]. This is consistent with these two CAR isoforms having different biological functions within a polarized epithelium. IL-8 also has an acute effect that stimulates maximal CAR^Ex8^ expression between 4–12 h (Fig. 1E), suggesting that CAR^Ex8^ might be crucial in facilitating early innate immunological responses. It is possible that prolonged IL-8-mediated signaling or apical CAR^Ex8^ expression would lead to excessive levels of neutrophils at the apical surface, and adverse immunological complications due to prolonged inflammation. Future work will focus on elucidating the effect of CAR^Ex8^ on inflammation and bacterial clearance, particularly in the presence of inflammatory diseases, such as CF.

We hypothesized that the endogenous biological function of CAR^Ex8^ at the apical epithelial cell surface is to tether infiltrating neutrophils transmigrating from the basolateral interstitial space. We report for the first time that stimulation of cells with IL-8 or overexpression of CAR^Ex8^ increases neutrophil adhesion at the apical surface of epithelia (Figs. 1H, 3 and S1). Consistent with a major role for apical CAR^Ex8^ in neutrophil adhesion to the apical surface, neutrophil binding could be blocked completely by AdV5 FK, but not by FK from a non-CAR binding AdV (Fig. 3D). Moreover, apical surface adhesion of CHO cells, normally lacking CAR and JAML expression, to polarized MDCK cells was significantly enhanced by over expression of CAR^Ex8^ or JAML. This indicates that cells expressing either adhesion molecule could adhere to apical CAR (S4B Fig). Future studies will compare the importance of CAR^Ex8^ to epithelial ICAM-1, which is the major rhinovirus receptor and has been identified as a neutrophil binding partner when epithelia are stimulated by IFNγ and TNFα [38,39]. Finally, we show that neutrophils transmigrating through the epithelium bind to the apical surface upon induction of CAR^Ex8^ (Fig. 4A). Taken together, an endogenous biological function of CAR^Ex8^ is to tether infiltrating neutrophils at the epithelial apical surface.

Enhanced adhesion of neutrophils to the epithelial apical surface may serve several important biological functions. For example, retention would prevent transmigrating neutrophils from being washed away into the airway lumen, and local retention in the region of IL-8 secretion would maintain focused inflammation that would prevent damage to neighboring regions. Retention would also allow neutrophils to achieve the critical concentration required to efficiently kill invading pathogens [20] and form a defensive barricade that prevents further infection of the epithelium. It is also possible that apical CAR^Ex8^ contributes to tight junction integrity as neutrophils break through to the apical surface. Each of these possibilities will be examined in future work.

Although neutrophils are normally expected to facilitate the clearance of microbial pathogens, we have discovered a new repercussion for the accumulation of neutrophils at the epithelial apical surface: adhered neutrophils enhance AdV infection (Fig. 5). We observed that upon addition of neutrophils there is nearly a log increase in the AdV5 FK-sensitive viral entry (Fig. 5B-E). This effect appears to be CAR^Ex8^ specific since an additional increase in AdV entry was not observed when the expression of CAR^Ex7^ or mCherry was turned on in MDCK-CAR^Ex7^ and MDCK-mCherry cells, respectively. We propose that AdV may have evolved to co-opt the innate immune response of the host in order to enhance entry into polarized epithelia. This is consistent with the fact that AdV early protein E1A stimulates the host cell to secrete IL-8 [6,40]. In addition, it is possible that under pathological conditions, such as CF, where excess neutrophils accumulate on the cell surface, AdV entry might be augmented even further. There could be several mechanisms by which the neutrophils might be promoting viral infection. For example, it is possible that the apically adhered neutrophils cause epithelial cell signaling which culminates in the loosening of the junctions to enable increased neutrophil recruitment [38]. This is not likely given that AdV infection is highly efficient from the basolateral surface and overexpression of basolateral CAR^Ex7^ does not enhance apical AdV infection (Fig. 5D). Moreover, there was no change in TER in the presence of adhered neutrophils (Fig. 5F). Neutrophils are known to secrete IL-8 [41] and therefore may stimulate apical CAR^Ex8^ synthesis and localization. While this would accommodate additional infiltrating neutrophils, AdV FK has greater affinity for CAR than neutrophil JAML and would be able to take advantage of the additional receptors to enter the epithelium. Apically adhered neutrophils may also release inflammatory mediators that alter fluid phase endocytosis from the apical surface to facilitate viral entry. Future experiments will focus on elucidating the exact mechanism(s) behind neutrophil-enhanced viral entry. Understanding this may lead to novel therapies to inhibit AdV infection or reduce the toxic effects of chronic inflammation.

Several major steps of the mechanisms underlying the IL-8-mediated increase in CAR^Ex8^ expression have been identified. IL-8 regulates CAR^Ex8^ expression by post-transcription mechanisms (Figs. 6 and S3). Other mechanisms, such as protein stabilization, an increase in the half-life of CAR^Ex8^, or enhanced trafficking to the apical surface, may also contribute to the effect of IL-8 and will be examined in the future. However, the reversal of the effect of IL-8 by protein synthesis inhibition and the effect of plasmid-expressed S6K, an enzyme known to directly upregulate the expression of several proteins by post-transcriptional mechanisms [28,42], demonstrate an important role for IL-8 signaling in *de novo* CAR^Ex8^ protein synthesis.

Our data show that IL-8 triggers the activation of AKT and activation of its proximal target, S6K. This is consistent with previous studies demonstrating that IL-8 signaling via AKT and S6K post-transcriptionally upregulates the protein synthesis of cyclin D1 [28,42]. In addition, consistent with our previous studies that show that GSK3β negatively regulates CAR^Ex8^ expression [16], we demonstrate that IL-8 signaling results in the inhibition of GSK3β (Fig. 6H) and upregulation of CAR^Ex8^ expression. To our knowledge, this is the first time that GSK3β inhibition has been shown to occur upon IL-8 exposure. Based on our data and the literature, GSK3β inhibition is most likely downstream of S6K activation [43]. Importantly, we demonstrated that the treatment of Calu-3 cells with IL-8 in the presence of AKT/S6K inhibitors decreases AdV entry, while the inhibition of GSK3β augments AdV entry.

**Fig 7:**
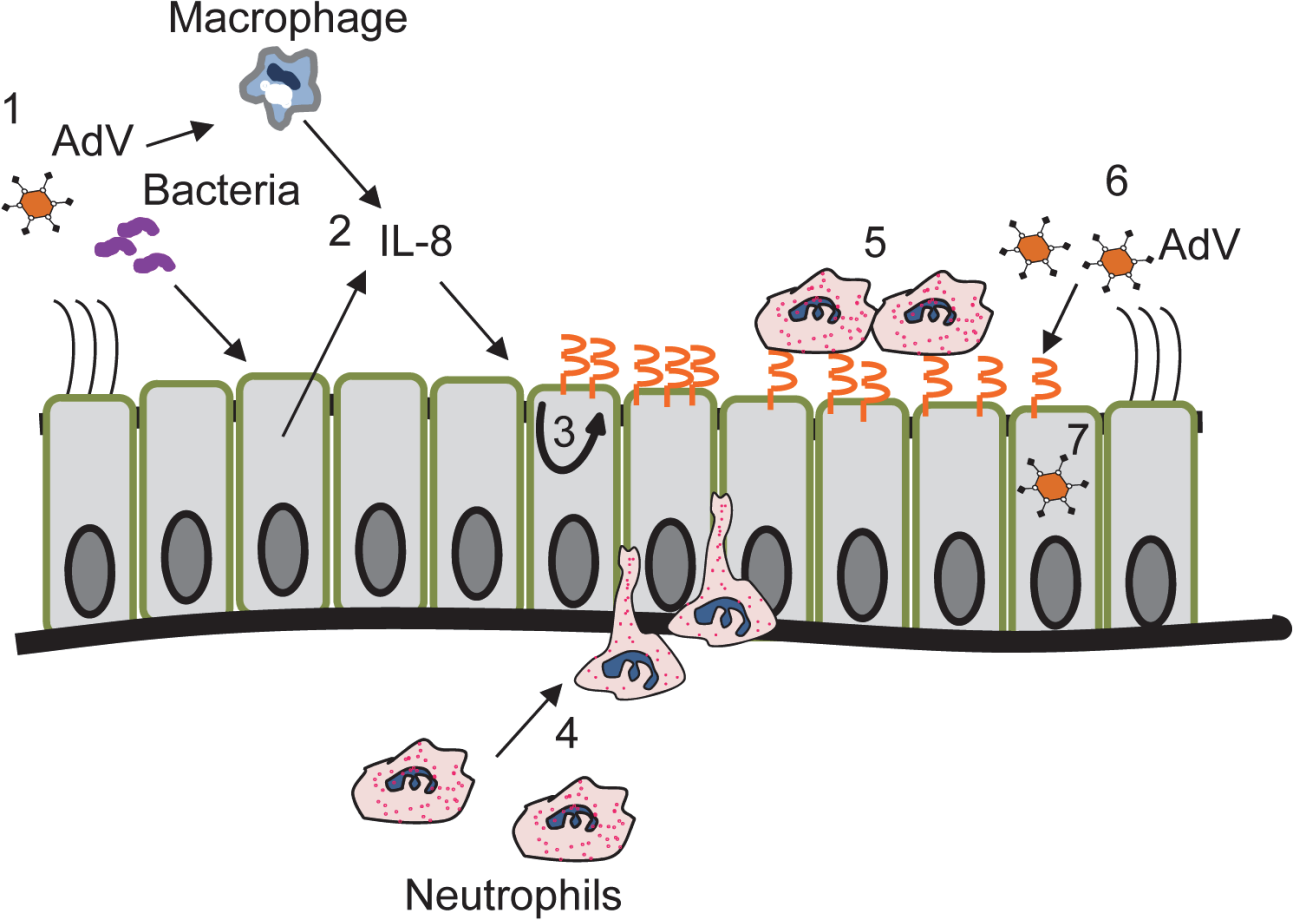
Schematic of IL-8-mediated enhancement of AdV entry into polarized epithelia. 1) Pathogenic microbes that invade the airway 2) cause both the resident macrophages and the epithelial cells to secrete IL-8. 3) IL-8 exposure causes intracellular signaling within the epithelial cells that augments *de novo* protein synthesis and apical localization of CAR^Ex8^. 4) IL-8 simultaneously recruits neutrophils that transmigrate through the epithelium from the basal surface to the apical surface and 5) bind to CAR^Ex8^ at the apical surface of the epithelium. 6) AdV entering the airway hijacks the host innate immune response and apical CAR^Ex8^ to gain entry into the host cell.

Taken together (Fig. 7), these data indicate that IL-8, potentially derived from stimulated resident macrophages or the epithelium itself, activates specific signaling pathways within polarized epithelial cells (Fig. 6L) that lead to increased apical CAR^Ex8^ and retention of transmigrating neutrophils at the apical surface. AdV has likely evolved to hijack this innate pathway and induced apical CAR^Ex8^ expression for entry into a polarized epithelium from the apical surface. Previously, it was assumed that AdV must breach the tight junction barrier to access its primary receptor. This study provides a novel mechanism and an explanation as to how the virus can infect the intact epithelium without breaching the barrier. Further elucidating these mechanisms in both healthy and diseased individuals will yield a greater understanding of the susceptibility of the airway epithelium to invading viral pathogens and interventions that reverse this effect. Moreover, if CAR^Ex8^ is upregulated in diseased conditions, novel therapies that target the CAR-neutrophil interaction may present a new anti-inflammatory treatment for inflammatory airway disease.

## Materials and Methods

### Ethics statement

Primary human airway tracheal epithelial cells were isolated from the lungs of healthy human donors under IRB approval by the Institutional Review Board of the University of Iowa (IRB ID No. 9507432) and according to the principles expressed in the Declaration of Helsinki. Primary human airway epithelia were isolated from discarded and de-identified trachea and bronchi of donor lungs and were analyzed anonymously. This study used discarded lung tissue, thus the IRB deemed consent was not needed.

### Cell culture and reagents

Calu-3 cells, cultured as described [16], or MDCK, cultured as described [44], were plated on 6 and 24 well dishes (Thermo Fisher Scientific), respectively, and allowed to polarize for at least 4 days, or 12 mm millicells (Millipore) with 0.4 μm pores for standard polarization experiments or with 3 μm pores for transmigration studies at a density of 2.5 X 10^5^ cells/millicell and grown at the air-liquid interface until TER was >600 Ω•cm^2^ as measured by a chopstick ohmmeter (World Precision Instruments, Sarasota, FL). Primary human airway tracheal epithelial cells were a kind gift from Dr. Joseph Zabner, University of Iowa Cells and Tissues Core, Iowa City, IA. Primary airway epithelial cells, cultured and expanded as described [45], were seeded on millicells and allowed to differentiate, as described [14,46], for >2 weeks and TER >600 Ω•cm^2^. Myc-tagged S6K was cloned into pRK5 plasmid under the CMV promoter. IL-8 was purchased from Gold Biotechnology (St. Louis, MO), cycloheximide was from Sigma, and all other inhibitors were from Tocris Bioscience (Bristol, United Kingdom). Plasmid for HA-tagged AdV5 FK was a kind gift from Dr. Glen Nemerow and Tina-Marie Mullen (The Scripps Research Institute, CA). Purified AdV3 FK was a kind gift from Dr. André Lieber (University of Washington, Seattle, WA). Total CAR (1605p) and CAR^Ex8^-specific (5678p) Abs have previously been described [44]. Ab for actin was from Sigma, E-cadherin from Life Technologies, FLAG from AbCam, AKT, S6K, GSK3β, and phosphospecific antibodies from Cell Signaling Technology (Danvers, MA).

### AdV5-β-Gal infection, viral entry, and β-galactosidase assay

Viruses were purchased from the University of Iowa Gene Transfer Vector Core. Epithelia were infected with recombinant AdV5-β-Gal at a multiplicity of infection (MOI) of 100 plaque forming units (pfu) per cell, or as indicated in the text, for 1 h at 37°C, washed with PBS, and lysed 24 h later for β-Gal protein expression and DNA isolation for qPCR for the AdV5 *hexon* gene, *GAPDH*, or MDCK *actin*, as previously described [16] and detailed Supplemental Experimental Procedures (S1 Text). AdV5-β-Gal is replication defective and the copy number of the AdV5 *hexon* gene 24 h post-infection is indicative of the total number of AdV5 genomes present in a cell. Consistent with previous studies [47], no significant amount of cell surface bound AdV5 is observed after 24 h, as measured by the trypsinization of virus off of epithelia prior to DNA extraction, indicating that the viral DNA isolated by this assay is within the epithelial cells (S4C Fig).

### Cell surface biotinylation and western blot analysis

Western blot analysis and cell surface biotinylation with Sulfo-NHS-SS-Biotin (Thermo Scientific) were performed as previously described [14–16] and as detailed in the Supplemental Experimental Procedures.

### Generation of MDCK stable cells

The Lenti-X Tet-On advanced inducible expression system was used according to the manufacturer’s protocol (Clontech Laboratories) and as detailed in Supplemental Experimental Procedures.

### Neutrophil adhesion and transmigration assay

MDCK-CAR^Ex8^,-CAR^Ex7^ and-mCherry cells, either mock- or DOX-induced, were polarized on a 24-well dish or on millicells, as above. Neutrophils were isolated, as described previously [48], from the peripheral blood of healthy donors who signed an Institutional Review Board-approved consent form. A neutrophil adhesion assay was performed as described [49]. Briefly, freshly isolated neutrophils from the peripheral blood of healthy human donors were stained with 1.5 μM calcein green for 30 min at 37°C. Stained neutrophils in 300 μl HBSS were added to the apical epithelial cell surface, spun down at 140 x g for 4 min without any centrifuge break, and allowed to adhere for 15 min at 37°C in a CO_2_ incubator prior to washing and imaging or addition of AdV5-β-Gal. Neutrophil binding to the apical surface of the epithelium is stable for 1–2 h after which they detach. After 1 h AdV5-β-Gal infection, epithelia are washed multiple times to ensure removal of neutrophils prior DNA extraction 24 h post infection. To block neutrophil adhesion or AdV5-β-Gal infection, the epithelial cells were incubated with either purified AdV5 FK or AdV3 FK for 10 min at room temperature prior to the addition of the neutrophils, as above, and washed 3 times with HBSS+/+ to remove the unbound neutrophils. Neutrophils were imaged using fluorescence microscopy (Nikon Eclipse TE 2000–5) and the fluorescence intensity was quantified using the Metamorph software program (Metamorph Meta Imaging Series 6.1). Polarized Calu-3 cells on the 24 well dish were treated with IL-8 (30 ng/ml) for 4 h and washed to remove IL-8 prior to neutrophil adhesion assay as described above.

Neutrophil transmigration assay was performed, as previously described [50], in the physiologically relevant basal-to-apical surface direction. Briefly, 10^6^ fluorescently-labelled neutrophils were added to the upper chamber (basolateral surface) of MDCK-stable cells polarized on millicells (3 μM pore) in an inverted fashion [51] and stimulated to migrate in response to 100 nM n-formyl-methionyl-leucyl-phenylalanine (fMLP; AbCam) added to the apical surface for 1 h at 37°C. Post-neutrophil transmigration, the neutrophils that successfully transmigrated to the bottom chamber (transmigrated neutrophils) were imaged with a fluorescence microscope. Apically-adhered neutrophils were detached, as previously described [49]. Briefly, millicells were transferred to a fresh 24-well dish, spun at 50 x g for 5 min, imaged and quantified as above.

### Statistical analysis

All experiments were performed in triplicate. Microsoft Excel, Graph Pad Prism V5, or SPSS were used to perform statistical analyses. Statistical significance was evaluated using ANOVA or t-test, as indicated.

## Supporting Information

S1 Fig IL-8 induces neutrophil adhesion on the surface of polarized Calu-3 cells. A) Polarized Calu-3 cells were either mock or IL-8 treated for 4 h followed by an adhesion assay with primary neutrophils stained with calcein green. A) Bound neutrophils were imaged by fluorescence microscopy (10X; white bar = 150 μm) and B) quantified using Metamorph software. Error bars represent the SEM from three independent experiments: **p < 0.001 by student’s t-test.
(TIF)

S2 Fig IL-8 does not affect CAR^Ex8^ mRNA levels but stimulates its protein synthesis in Calu-3 cells. A) The apical surfaces of polarized Calu-3 cells were treated with increasing concentrations of IL-8 for 4 h before mRNA was isolated, cDNA synthesized, and analyzed for changes in the gene expression relative to GAPDH using qPCR analysis. Error bars represent standard error of the mean (SEM) from three independent experiments. No significant difference was found by one-way ANOVA. B) Polarized Calu-3 cells were treated with IL-8 in the presence or absence of cycloheximide (CHX) for 4 h. and cell lysates analyzed for CAREx8 and actin protein expression.
(TIF)

S3 Fig Quantitation of the blots in Fig. 6. A) Treatment of airway epithelial cells with IL-8 results in a significant increase in the expression of CAR^Ex8^ which is inhibited by the protein synthesis inhibitor CHX. IL-8 also increased the levels of B) pAKT-T308 and C) pS6K T389. The IL-8-mediated increase in CAR^Ex8^ is blocked by the AKT and S6K inhibitors D) Ly294002 and E) R0318220, respectively. F) Overexpression of Myc-S6K plasmid resulted in the significant increase in the expression of CAR^Ex8^ which was further stimulated in the presence of IL-8. G) IL-8 increased the levels of pGSK3βS9. H) Treatment of airway epithelial cells with GSK3β inhibitor SB415286 and LiCl stimulated CAR^Ex8^ protein expression. I) IL-8 in the presence of both S6K and GSK3β inhibitors, RO318220 and SB415286, cause a significant increase in CAR^Ex8^ expression. Error bars represent the SEM from three independent experiments: *p < 0.05 by student’s t-test or one-way ANOVA and Bonferroni post hoc test.
(TIF)

S4 Fig A) Polarized Calu-3 cells were either untreated or treated with IL-8 for 4 h followed by apical cell surface biotinylation to examine the protein expression of Integrin β1 and CAR^Ex8^. Whole cell lysate was probed with actin to demonstrate equal loading. B) CHO cells were mock transfected (CHO) or transfected with CAR^Ex8^ or JAML and labeled with calcein green for an adhesion assay on polarized MDCK cells as described for neutrophils. C) Polarized MDCK-CAR^Ex8^ cells were uninfected or infected with AdV5-β-Gal for 1 h and untreated or treated with trypsin before DNA isolation 24 h post infection. qPCR analysis for AdV5 *Hexon* is relative to uninfected cells.
(TIF)

S1 Text Supplemental methods. (DOCX)
