# Supplementary figures and images for: Adenovirus Entry From the Apical Surface of Polarized Epithelia Is Facilitated by the Host Innate Immune Response
Source: PLoS Pathog. 2015 Mar 13;11(3):e1004696. doi: 10.1371/journal.ppat.1004696 (PMC4358923; doi:10.1371/journal.ppat.1004696)

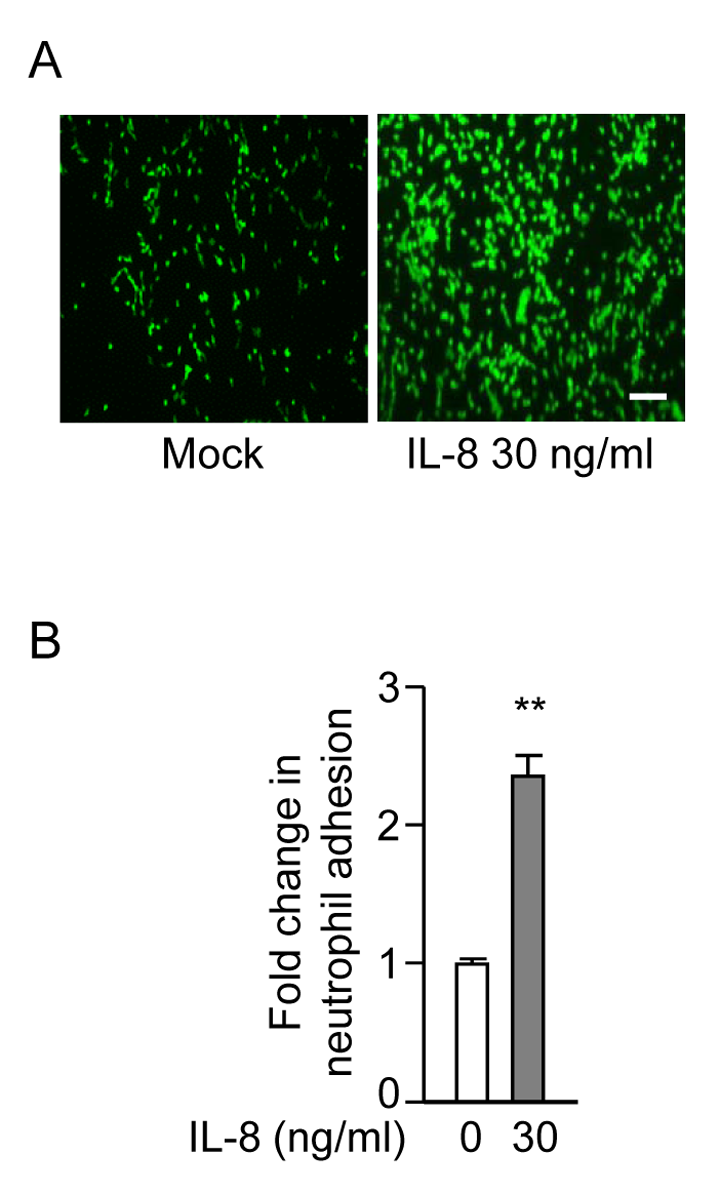

Supplement: S1 Fig — A) Polarized Calu-3 cells were either mock or IL-8 treated for 4 h followed by an adhesion assay with primary neutrophils stained with calcein green. A) Bound neutrophils were imaged by fluorescence microscopy (10X; white bar = 150 μm) and B) quantified using Metamorph software. Error bars represent the SEM from three independent experiments: **p < 0.001 by student’s t-test. (TIF) [file ppat.1004696.s001.tif]

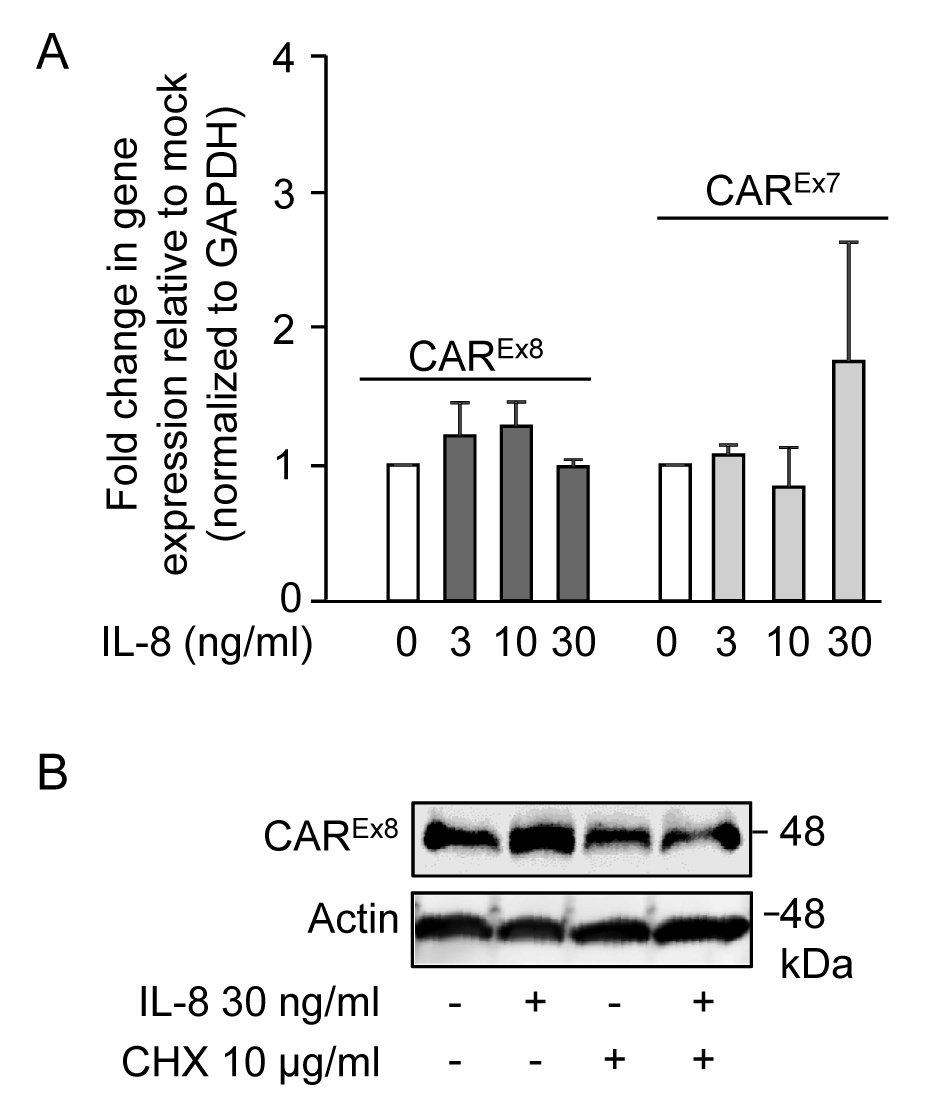

Supplement: S2 Fig — A) The apical surfaces of polarized Calu-3 cells were treated with increasing concentrations of IL-8 for 4 h before mRNA was isolated, cDNA synthesized, and analyzed for changes in the gene expression relative to GAPDH using qPCR analysis. Error bars represent standard error of the mean (SEM) from three independent experiments. No significant difference was found by one-way ANOVA. B) Polarized Calu-3 cells were treated with IL-8 in the presence or absence of cycloheximide (CHX) for 4 h. and cell lysates analyzed for CAREx8 and actin protein expression. (TIF) [file ppat.1004696.s002.tif]

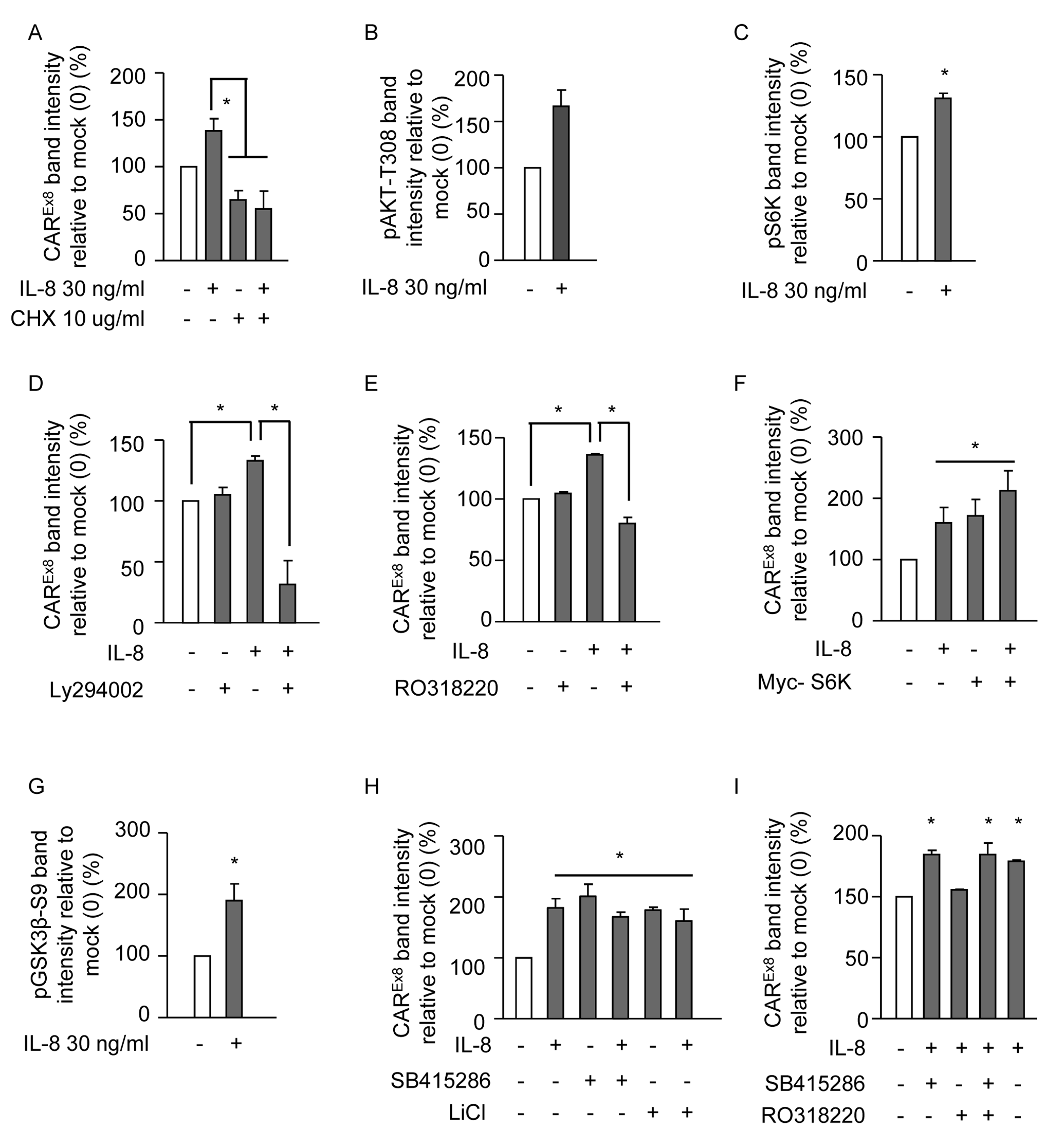

Supplement: S3 Fig — A) Treatment of airway epithelial cells with IL-8 results in a significant increase in the expression of CAREx8 which is inhibited by the protein synthesis inhibitor CHX. IL-8 also increased the levels of B) pAKT-T308 and C) pS6K T389. The IL-8-mediated increase in CAREx8 is blocked by the AKT and S6K inhibitors D) Ly294002 and E) R0318220, respectively. F) Overexpression of Myc-S6K plasmid resulted in the significant increase in the expression of CAREx8 which was further stimulated in the presence of IL-8. G) IL-8 increased the levels of pGSK3βS9. H) Treatment of airway epithelial cells with GSK3β inhibitor SB415286 and LiCl stimulated CAREx8 protein expression. I) IL-8 in the presence of both S6K and GSK3β inhibitors, RO318220 and SB415286, cause a significant increase in CAREx8 expression. Error bars represent the SEM from three independent experiments: *p < 0.05 by student’s t-test or one-way ANOVA and Bonferroni post hoc test. (TIF) [file ppat.1004696.s003.tif]

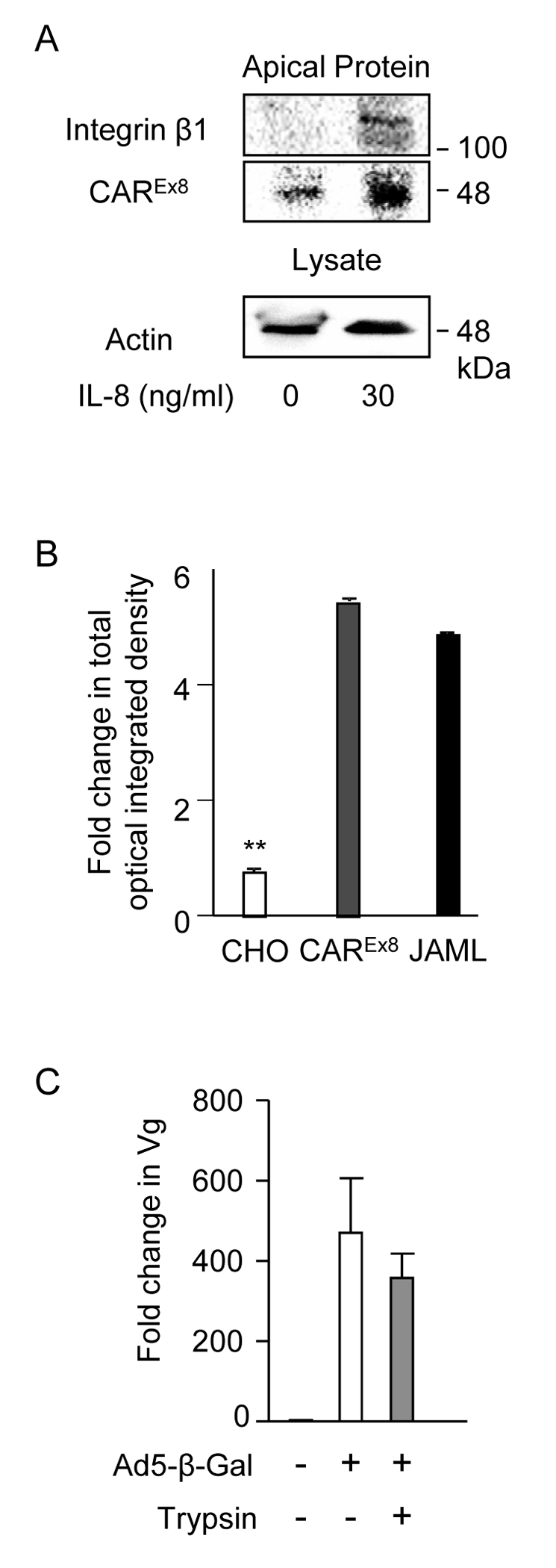

Supplement: S4 Fig — Whole cell lysate was probed with actin to demonstrate equal loading. B) CHO cells were mock transfected (CHO) or transfected with CAREx8 or JAML and labeled with calcein green for an adhesion assay on polarized MDCK cells as described for neutrophils. C) Polarized MDCK-CAREx8 cells were uninfected or infected with AdV5-β-Gal for 1 h and untreated or treated with trypsin before DNA isolation 24 h post infection. qPCR analysis for AdV5 Hexon is relative to uninfected cells. (TIF) [file ppat.1004696.s004.tif]
