# Supplementary material for: Adenovirus Entry From the Apical Surface of Polarized Epithelia Is Facilitated by the Host Innate Immune Response
Source: PLoS Pathog. 2015 Mar 13;11(3):e1004696. doi: 10.1371/journal.ppat.1004696 (PMC4358923; doi:10.1371/journal.ppat.1004696)
Supplement: S1 Text — (DOCX) [file ppat.1004696.s005.docx]

**Supplemental Information**

**Adenovirus entry from the apical surface of polarized epithelia is facilitated by the host innate immune response**

Poornima L.N. Kotha, Priyanka Sharma, Abimbola O. Kolawole, Ran Yan, Mahmoud S. Alghamri, Trisha Brockman, Julian Gomez-Cambronero, and Katherine J.D.A. Excoffon

**Text S1. Supplemental Methods**

**Generation and characterization of MDCK stable cells:** cDNAs encoding FLAG-tagged human CAR^Ex8^, FLAG-tagged human CAR^Ex7^, or mCherry were cloned into the pLVX-tight-puro vector using Infusion Cloning (Clontech Laboratories). pLVX-Tet-on advanced and each pLVX-tight-puro plasmid were packaged separately into the lentivirus by transfecting the plasmids into the 293T packaging cell line along with the Lenti-X HTX packaging system using the manufacturer’s protocol (Clontech Laboratories). MDCK cells were first infected with the pLVX-Tet-on lentivirus in the presence of neomycin antibiotic drug (G418) that selects for Tet-on transduced cells. Neomycin resistant cells were used for further selection by serial dilution into a 96-well plate such that there was only one cell per well. The neomycin resistant clones from single cells were expanded and screened for the stable expression of pLVX-Tet-on, morphology similar to parental cells, and the ability to form an epithelium as measured by TER. A single MDCK-Tet-on cell line was transduced with each of the pLVX-tight-puro lentiviruses carrying our genes of interest in the presence of puromycin (a drug that selects for the pLVX-tight-puro transduced cells) and neomycin. As above, clones from single cells were selected and screened for stable integration of FLAG-CAR^Ex8^, FLAG-CAR^Ex7^, or mCherry by PCR with a primer sets that detected the exogenous but not the endogenous CAR^Ex8^ and CAR^Ex7^. To accomplish this specificity, the forward and the reverse primer used to detect exogenous CAR^Ex7^ overlapped the junction between exon 6 and exon 7 for the upstream primer while the downstream primer was in the CAR^Ex7^-specific sequence (CAR-F: 5’ GTCCCTCCTTGAAATAAAGCTG; CAR7-R: 5’ CGGATCCCTATACTATAGACCCATC). PCR for CAR^Ex8^ used the same upstream primer but the downstream primer overlapped the splice site between exon 7 and exon 8 (CAR8-R: 5’ GTGGATCCTTATACAACTGTAATTCCA). Using such primers eliminates the possibility of detecting the endogenous CAR^Ex7^ or CAR^Ex8^ gene in which the exons are interspersed with long introns. Primers for mCherry were PmCherry-F: 5’ GAATTCGCCACCATGGTGAGC, PmCherry-R2 5’ ATCGATTCACTTGTACAGCTCG. At least 5 clones derived from single cells for each gene of interest were compared to parental MDCK and MDCK-Tet-on to identify clones with similar characteristics, in the absence of DOX, including: growth rate, as measured by doubling time; polarization characteristics, including the ability to form tight junctions, as measured by TER, distribution of total CAR (1605p), CAR^Ex8^, apical protein (Ezrin), basolateral proteins (E-cadherin, β-catenin), and tight junction protein (ZO-1), by immunocytochemistry, cell surface biotinylation, and Western blot. Polarity of baseline AdV5-β-Gal entry and transduction were measured in cells polarized on millicell inserts by qPCR and β-Gal assay.

**Western Blotting**

For experiments involving IL-8, polarized epithelial cells were serum starved for 4 h prior to IL-8 treatment. To inhibit protein synthesis, cycloheximide (10 µg/ml) was added to the apical surface of polarized epithelial cells for 30 min prior to IL-8 treatment, maintained throughout the 4 h IL-8 treatment period, and then washed off. For experiments involving MDCK cells, the polarized cells were either uninduced or induced with DOX, as indicated in the text, for 24 h. Post-treatment, cells were washed with ice-cold PBS, and lysed in buffer (50 mM Tris pH 7.4, 137 mM NaCl, 1% Triton X-100, 1 mM Na_2_VO_4_, 10 µg/ml protease inhibitors (leupeptin, aprotinin, pepstatin), and 1 mM phenylmethylsulfonyl fluoride) by rocking at 4°C. Cells were scraped, sonicated five times with five pulses and centrifuged at 17,000 x g for 10 minutes in a microcentrifuge. The supernatant was transferred to fresh tubes and protein concentration was determined with the Bio-Rad protein assay (Bio-Rad). Equal amounts of protein were subjected to 10% polyacrylamide gel electrophoresis. Gels were transferred to a polyvinylidene difluoride (PVDF) membrane (Millipore, Bedford, MA), blocked with 5% BSA, washed, probed with primary antibodies as indicated in the text, washed and incubated with HRP-conjugated secondary antibodies (Jackson Immuno Research). Band detection with ECL reagents (Pierce) was imaged on a Fuji LAS 4000. All blots are a representative from a minimum of three individual experiments.

**Cell surface biotinylation:** After IL-8 treatment for 4 h, polarized cells were treated with 1 mg/ml Sulfo-NHS-SS-Biotin (Thermo Scientific) for 1 h at 4°C with rocking, washed with ice cold PBS, and then any remaining free Sulfo-NHS-SS-biotin was quenched with ice cold 100 mM glycine for 20 min at 4°C. The cells were then washed three times with PBS including Ca^2+^ and Mg^2+^ (PBS +/+) and lysed with lysis buffer as described above. NeutrAvidin beads (Thermo Scientific) were added to the clarified cell lysate and incubated at 4°C for 2 h with rotation. The NeutrAvidin beads were then collected by centrifugation at 1,000 g at 4°C for 3 min and washed three times with ice-cold wash buffer. The Sulfo-NHS-SS-Biotin-labeled proteins were eluted from the NeutrAvidin beads with SDS-PAGE sample buffer at 65°C for 10 min. This was followed by SDS-PAGE and Western blotting using appropriate antibodies.

**RNA isolation, reverse transcription, real-time PCR, and AdV genome quantitation**

To investigate changes in gene expression, total RNA was isolated from polarized epithelial cells after treatment with IL-8 as indicated in the text and figures. Cells were lysed using TRIzol (Life Technologies) according to manufacturer’s protocol. cDNA was synthesized from 1 mg of RNA using the Quanta First Strand Kit (Quanta Bio Sciences) according to the manufacturer’s instructions prior to quantitative PCR (qPCR). For AdV5 genome quantification, 24 h post infection, cells were lysed and total DNA was purified using the DNeasy Blood and Tissue kit according to the manufacturer’s instructions (QIAGEN, Valencia, CA). Breifly, the cells were lysed in the presence of proteinase K. The lysate was added to the provided column to enable DNA binding. DNA was then eluted using 100 µl of Qiagen AE elution buffer. qPCR was performed using SYBRG with low ROX (Quanta) in a Stratagene Real Time PCR System (Agilent Technologies) using primers for AdV *hexon*, *glyceraldehyde 3-phosphate dehydrogenase* (*GAPDH*) or *β-actin* as internal standards. The relative expressions of target genes were quantified using comparative Ct analysis by using Mx4000p V5 software for data analysis. Primers used were: CAR-qPCR-F: 5’ TCGGCAGTAATCATTCATCCCTGG CAR^Ex7^-qPCR-R: 5′ ATAGACCCATCCTTGCTCTGTGCT, CAR^EX8^-qPCR-R: 5’ ACTGTAATTCCATCAGTCTTGTAAGGG; AdV hexon gene specific primers Ad-qPCR-F: 5’ CGCCTCGGAGTACCTGAG and Ad-qPCR-R: 5’ GTGGGGTTTCTGAACTTGT. Abundance relative to GAPDH gene expression was calculated for each gene of interest in human cells. GAPDH-F: 5’ CACCCTGTTGCTGTAGCCAAA, GAPDH-R: 5’ CAACAGCGACACCCACTCCT. In dog cells, CAR and AdV genomes were normalized to MDCK-Actin-F: 5’ AAGATCTGGCACCACACCTTCT-AC, MDCK-Actin-R: 5’ ATCTGGGTCATCTTCTCACGGTTG**.**
